# When does a melanoma metastasize? Implications for management

**DOI:** 10.18632/oncotarget.28591

**Published:** 2024-06-13

**Authors:** John F. Thompson, Gabrielle J. Williams

**Affiliations:** ^1^Melanoma Institute Australia, The University of Sydney, Sydney, NSW, Australia; ^2^Faculty of Medicine and Health, The University of Sydney, Sydney, NSW, Australia; ^3^Department of Melanoma and Surgical Oncology, Royal Prince Alfred Hospital, Sydney, NSW, Australia; ^4^Faculty of Health and Medical Sciences, University of Western Australia, Perth, WA, Australia

**Keywords:** melanoma, metastasis, time, adjuvant systemic therapy, tumor doubling time

## Abstract

Selecting which patients with clinically-localized melanoma require treatment other than wide excision of the primary tumor is based on the risk or presence of metastatic disease. This in turn is linked to survival. Knowing if and when a melanoma is likely to metastasize is therefore of great importance. Several studies employing a range of different methodologies have suggested that many melanomas metastasize long before the primary lesion is diagnosed. Therefore, waiting for dissemination of metastatic disease to become evident before making systemic therapy available to these patients may be less effective than giving them post-operative adjuvant therapy initially if the metastatic risk is high. The identification of these high-risk patients will assist in selecting those to whom adjuvant systemic therapy can most appropriately be offered. Further studies are required to better identify high-risk patients whose primary melanoma is likely to have already metastasized.

## INTRODUCTION

It is generally believed that early removal of a primary cutaneous melanoma will improve the patient’s outcome. There have been some studies that appear to support this concept [1–3] and it is consistent with the logical assumption that metastasis to lymph nodes and distant sites is less likely to occur if the primary tumor is removed expeditiously. However, available evidence suggests that melanoma metastasis has often occurred many months before a primary melanoma diagnosis is made. This implies that the patient’s long-term prognosis is unlikely to be influenced to any great extent by the interval between diagnosis and wide excision of the primary tumor, which is usually a few weeks at most.

### Why understanding the timing of melanoma metastasis is important

An understanding of when melanoma metastasis occurs is also important for several reasons other than the timing of surgery to widely excise the primary melanoma. Perhaps most importantly today, the concept of early metastasis provides the rationale for adjuvant systemic therapy after wide surgical excision for patients with higher-risk primary melanomas. It assumes that in some, possibly many, patients undiagnosed micrometastatic disease is already present in regional nodes or at distant sites, and that it can be eliminated by the systemic therapy. Supporting this proposition are the results of recent clinical trials which have demonstrated that outcomes for patients with resected Stage IIB and IIC melanomas are indeed improved if modern adjuvant systemic therapy (an immune checkpoint inhibitor such as pembrolizumab or nivolumab) is given [4, 5]. Another reason why understanding the time of melanoma metastasis is useful is that in medico-legal cases when a melanoma diagnosis has initially been missed and metastatic melanoma is subsequently discovered, an opinion is often sought as to when the melanoma is likely to have metastasized. The key question that lawyers, patients and relatives wish to have answered is whether earlier removal of the primary melanoma could have prevented potentially fatal metastasis? In other words, was a physician’s failure to diagnose a melanoma at the earliest possible time and arrange prompt treatment likely to have been responsible for an adverse outcome?

### Initiation and growth of metastases

The first phase of metastasis, namely release of tumor cells from a primary lesion, is reported to be highly efficient [6, 7], but the second phase, metastasis initiation, is not [7–9] and a single cell reportedly has a very low probability of successfully seeding from a primary tumor and becoming a viable metastasis, of the order of one in 10^8^ [10]. Thus, relative to the large number of cells that may disseminate from a primary tumor, only very few successfully form distant metastases [7, 11]. So theoretically, the more tumor cells that are present before surgical excision the more likely it is that successful metastasis will occur.

Early studies suggested that three fundamental principles apply to the growth rate of human tumors: (i) it is constant for long periods, (ii) it is often slow, and (iii) rates vary with different histological tumor types [12]. These principles have been repeatedly confirmed [13].

The increase in volume of a metastatic tumor is exponential, therefore it may appear to enlarge very quickly once it becomes clinically evident. By this time, usually with a metastatic deposit measuring 5–10 mm in diameter, there are an estimated 2.5×10^8^ tumor cells at the site, assuming an average cell diameter of 16 μm and a volume of 4×10^9^ cm^3^ [14]. To reach this size approximately 28 cell doublings would need to have occurred.

### Tumor doubling time

The concept of tumor doubling time (TDT) as a measure of metastatic melanoma growth was first proposed in the mid 1960s [15, 16]. It was originally estimated on the basis of changes in the size of lung metastases on sequential chest X-rays [16–18]. Reported studies showed a very wide range of TDT values, ranging from 8–118 days, with a median of 49 days for 17 reported estimates. Importantly, however, in individual patients the TDT of metastatic melanoma cells appeared to be constant [13, 19, 20]. That the TDT of melanoma cells can be long is demonstrated by the occasionally very late clinical appearance of metastases, sometimes 10, 20 or even 30 years after the primary melanoma was excised [21–24].

### Time of melanoma metastasis

**Figure 1:**
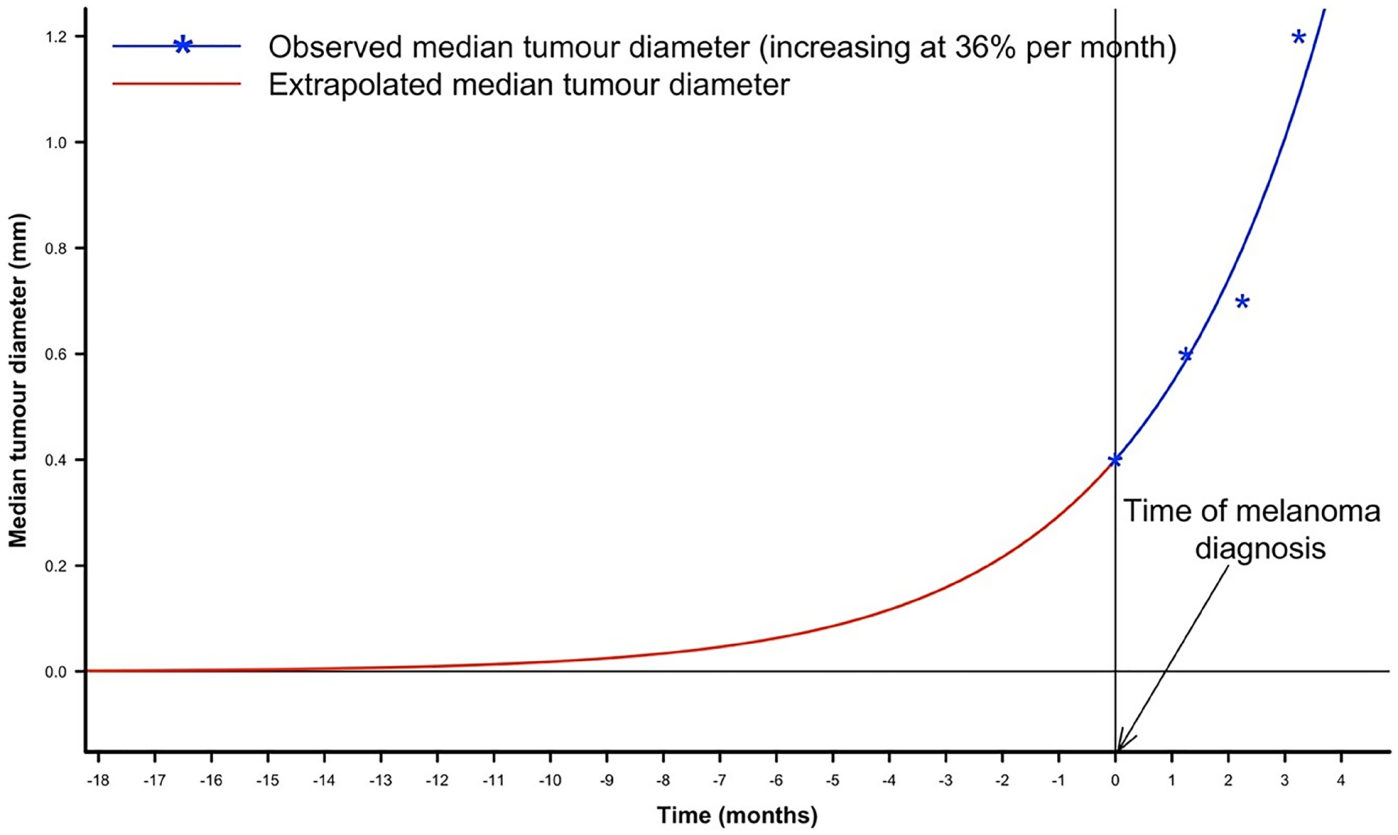
Backwards extrapolation of median sentinel node tumor diameter prior to the time of primary melanoma diagnosis in a population-based cohort of 1027 Dutch melanoma patients (reproduced with permission from *European Journal of Cancer* 167: 133–141, 2022 [25]).

There is extensive and compelling evidence that metastasis to regional lymph nodes and distant sites frequently occurs long before a primary melanoma is diagnosed. Based on the rate of growth of metastases and accepting that the TDT is likely to be constant in an individual patient, it is possible to estimate when metastasis first occurred. For example, in a study based on observations of the size of sentinel lymph node metastases at 1, 2 and 3 months after melanoma diagnosis, backwards extrapolation indicated that the first metastatic cell reached the sentinel lymph node approximately 18 months before the primary melanoma was diagnosed [25] (Figure 1). A study of 37 cases of uveal melanoma that progressed to disseminated disease indicated that growth of the first systemic metastasis commenced around 5 years before the primary lesion was recognized and treated [20]. Both the above studies confirm an earlier finding in 18 patients with lung metastases from melanoma that similarly concluded that metastatic growth began long before the primary melanoma was excised [26].

This pattern of metastatic initiation occurring long before primary diagnosis is not limited to melanoma; a similar conclusion was reached in studies of 337 cases of breast cancer with axillary lymph node metastases [27], 11 patients with lung metastases from sarcoma [28] and 110 cases of lung metastases from various tumor types [12, 15].

### Patterns of metastatic spread

In melanoma patient’s metastasis to regional lymph nodes via lymphatics is the most common form of spread, with around 50% of those who develop metastases having nodal disease as their first site of clinically-detected recurrence [29]. However, metastasis in a distant organ with no evidence of previous or current lymph node disease is seen in about 30% of those who develop metastatic melanoma [30, 31] suggesting dissemination of malignant cells exclusively via the bloodstream. Lymph node metastases are often diagnosed earlier than metastases at distant sites, with a median interval of 16 months between primary diagnosis and the detection of nodal metastasis in one study, while distant metastases tend to be detected a median of 25–40 months after primary diagnosis [29, 30]. This pattern suggests that bloodstream spread likely occurs later than lymphatic spread. It could be due to secondary dissemination from lymph node metastases, but this would not explain the 30% of patients with visceral melanoma metastases who never develop regional lymph node metastases. It is very likely that both routes of metastatic dissemination commonly occur, i.e., secondarily from a lymph node metastasis as well as directly from a primary lesion. The concept that both processes probably occur is supported by the results of genomic studies analyzing multi-site metastasis samples within individual patients. These have shown lineage diversification across different metastatic sites, indicating that some metastases probably developed from a prior metastatic site while others may represent separate metastatic seeding from the primary lesion [32].

### Identifying patients at risk of metastasis

At the time of a primary melanoma diagnosis it is possible to use clinico-pathological parameters to predict the likelihood of sentinel lymph node metastasis [33, 34] and of eventual death from melanoma [35] (which is usually due to systemic metastasis [36]). The most informative prognostic features for survival are Breslow thickness, ulceration and mitotic rate. These are widely used to identify higher risk patients and offer additional management such as sentinel node biopsy and systemic therapies. Nomograms predicting recurrence and survival are available [37, 38], but with further refinement based on genomics and/or proteomics it may be possible to more precisely predict patients whose melanoma has or will metastasize and identify more accurately the group most likely to benefit from systemic therapies.

## CONCLUSIONS

With a considerable body of evidence indicating that metastasis from a primary cutaneous melanoma often occurs long before the melanoma is diagnosed, the rationale for treating patients with high-risk primaries (i.e. those with Stage IIB and IIC melanomas) is very strong. Although it is yet to be definitively confirmed, it seems that immunotherapy is more likely to be effective at eliminating metastatic disease if the tumor burden is low, making it more logical to treat patients with high risk melanomas at the earliest possible time, rather than treating those who develop metastatic disease with the same drugs later, when the metastases have progressed from micrometastases to become secondary tumors able to be diagnosed clinically or by imaging.
